# Concepts and mechanisms underlying chemotherapy induced immunogenic cell death: impact on clinical studies and considerations for combined therapies

**DOI:** 10.18632/oncotarget.6113

**Published:** 2015-10-14

**Authors:** Simon Gebremeskel, Brent Johnston

**Affiliations:** ^1^ Department of Microbiology & Immunology, Dalhousie University, Halifax, Nova Scotia, Canada; ^2^ Department of Pediatrics, Dalhousie University, Halifax, Nova Scotia, Canada; ^3^ Department of Pathology, Dalhousie University, Halifax, Nova Scotia, Canada; ^4^ Beatrice Hunter Cancer Research Institute, Halifax, Nova Scotia, Canada

**Keywords:** cancer therapy, immunostimulation, immunogenic cell death, immunotherapy, chemotherapy

## Abstract

Chemotherapy has historically been thought to induce cancer cell death in an immunogenically silent manner. However, recent studies have demonstrated that therapeutic outcomes with specific chemotherapeutic agents (e.g. anthracyclines) correlate strongly with their ability to induce a process of immunogenic cell death (ICD) in cancer cells. This process generates a series of signals that stimulate the immune system to recognize and clear tumor cells. Extensive studies have revealed that chemotherapy-induced ICD occurs via the exposure/release of calreticulin (CALR), ATP, chemokine (C–X–C motif) ligand 10 (CXCL10) and high mobility group box 1 (HMGB1). This review provides an in-depth look into the concepts and mechanisms underlying CALR exposure, activation of the Toll-like receptor 3/IFN/CXCL10 axis, and the release of ATP and HMGB1 from dying cancer cells. Factors that influence the impact of ICD in clinical studies and the design of therapies combining chemotherapy with immunotherapy are also discussed.

## INTRODUCTION

Historically, the anti-cancer benefits of chemotherapies were considered to be a consequence of direct cytotoxicity or permanent arrest of the cell cycle machinery. These therapies were thought to non-specifically target rapidly proliferating cells, leading to the assumption that chemotherapies would inadvertently target proliferating immune cells and result in immunosuppression. Furthermore, chemotherapies were thought to induce cell death in an immunologically silent manner. This led many researchers to neglect the role of the immune system in cytotoxic chemotherapy, and the testing guidelines set by regulatory agencies recommended the use of immunodeficient hosts for examination of drug effects on cancer cells [1].

Regulated cell death is a physiological phenomenon that plays an important role in development and homeostasis [2, 3]. This process was initially characterized based on morphological cell changes, chromatin condensation, and membrane blebbing, and was thought to occur in the absence of an inflammatory response. Recently, a consensus has emerged recommending that the nature of cell death should not simply be defined by morphological changes, but rather by distinct molecular, biochemical, and metabolic hallmarks [2, 4]. This has led to the acceptance of a new apoptotic cell death modality that elicits antigen specific immune responses against dead-cell antigens [5]. This type of cell death has been termed immunogenic cell death (ICD) and was initially characterized in the context of anti-cancer chemotherapy [6].

Unlike physiological cell death which induces signals that lead to tolerogenic clearance of cells, chemotherapy-induced cell death generates specific changes in cell surface structures and release of soluble mediators that allow dendritic cells (DCs) to detect the dying cell and initiate an anti-tumor immune response [7]. During this process, DCs engulf parts of the stressed/dying cell and incorporate antigenic peptides into MHCs for presentation to T cells. In contrast to tolerogenic cell clearance, it is essential that DCs engulfing dying cells also receive maturation signals via cytokines or Toll-like receptor (TLR) signaling in order to activate T cells optimally and prevent the development of tolerance [8-10].

While many chemotherapeutics do not elicit ICD (e.g. etoposide, mitomycin C, cisplatin), some agents (including anthracyclines and oxaliplatin) have been shown to cause ICD [6, 11-14]. Additional therapeutic modalities that have been shown to induce ICD include radiation therapy [15, 16], oncolytic virus therapy [17, 18] and photodynamic therapy [19]. This review will focus on mechanisms of chemotherapy induced ICD. The only way to identify bona fide ICD inducers is through vaccination challenges [20]; tumor cells treated with ICD inducers prior to inoculation into immunocompetent mice protect mice from subsequent challenge with the same tumor [6, 11, 21, 22]. After screening for proteins that are upregulated on the surface of cancer cells undergoing ICD, the endoplasmic reticulum (ER) protein calreticulin (CALR) exhibited increased translocation to the plasma membrane [22]. The exposed CALR (ecto-CALR) was found to be critical for ICD as knockdown of CALR expression significantly hampered anti-tumor immunity [16, 22]. Following the pre-apoptotic ecto-CALR mobilization, cells undergoing ICD release ATP, which is essential for recruitment of antigen presenting cells (APCs) and the subsequent activation of the inflammasome to promote IL-1β release by DCs [12, 21, 23-25]. Anthracycline treated cancer cells also upregulate a TLR3/type I IFN/chemokine (C–X–C motif) ligand 10 (CXCL10) signaling cascade that results in protection from tumor growth [26]. In the late stages of apoptosis (secondary necrosis), cells passively release high-mobility group box 1 (HMGB1) which signals via TLR4 on DCs to enhance antigen presentation [11, 27, 28]. These processes are discussed in detail below.

## ER STRESS AND CALR EXPOSURE

The ER serves as a site for protein folding, modification, and trafficking. In addition, the ER is also the primary site for lipid biosynthesis and calcium storage. Physiologic stress such as increased secretory load, or pathologic stresses such as mutated proteins, can overwhelm the functional capacity of the ER, leading to ER stress [29]. The ER responds to stress by activating an adaptive mechanism called the unfolded protein response (UPR) [29]. The UPR evokes coping mechanisms such as expansion of the ER, attenuation of protein translation, reduced translocation of proteins to the ER, increased synthesis of chaperones, and increased protein degradation via the 26S proteasome [29]. The main purpose of these processes is to re-establish homeostasis and promote survival. However, when these coping mechanisms are overwhelmed, the pro-survival mechanisms switch to pro-death signals [30]. This is accompanied by translocation of danger signals to the cell surface and intrinsic mitochondrial apoptosis [31-33]. CALR represents the most abundant protein in the ER lumen and gets translocated to the surface of stressed and dying cancer cells [22, 34, 35]. This translocation of CALR occurs prior to translocation of phophatidylserine (PS) to the outer leaflet of the plasma membrane, hence it is termed a pre-apoptotic event [22, 34, 35]. The ecto-CALR serves as a potent “eat me” signal for local patrolling DCs.

**Figure 1:**
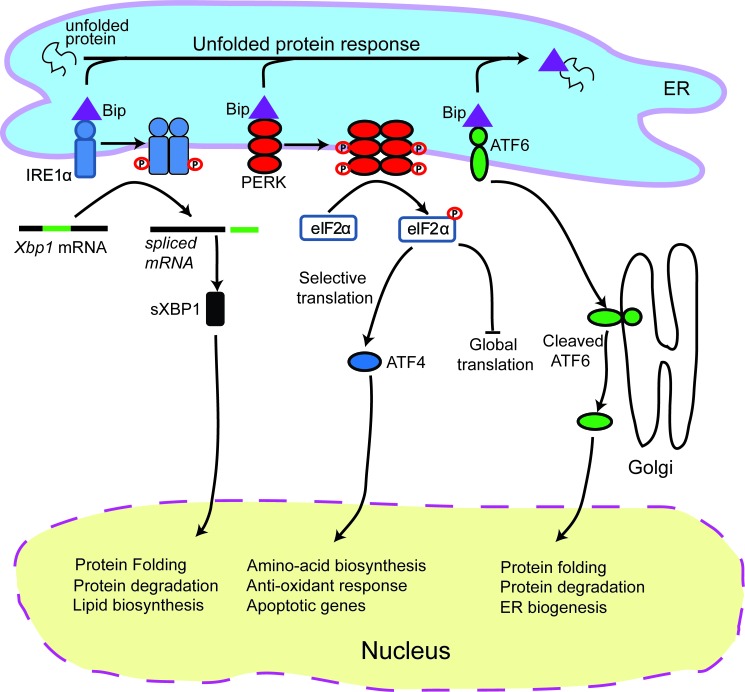
Schematic of the unfolded protein response Endoplasmic reticulum (ER) stress triggers PERK activation. Activated PERK attenuates protein biosynthesis by phosphorylating eIF2α which halts global translation, but leads to activation of ATF4. ER stress also triggers IRE1α activation which initiates the splicing of XBP1 mRNA, producing an active transcription factor, sXBP1. This leads to the expression of chaperone proteins and proteins involved in protein degradation. In addition, ER stress also activates ATF6 which also increases chaperone synthesis to alleviate ER stress.

The UPR is activated by three distinct sensors: the inositol-requiring enzyme 1 (IRE1) (an ER kinase and endoribonuclease) [36, 37], activating transcription factor 6 (ATF6) [38, 39] and PKC related kinase-like ER kinase (PERK) (Figure 1) [40, 41]. Under homeostatic conditions, the three UPR sensors are held in an inactive state by the chaperone binding immunoglobulin protein (BiP; also known as GRP78) [39, 42]. During stress, BiP preferentially associates with misfolded proteins to prevent them from aggregating, allowing the three UPR sensors to be activated. Activated ATF6 dissociates from the ER and translocates to the Golgi, where it is cleaved. Cleaved ATF6 acts as a transcription factor and translocates to the nucleus where it binds the ER stress response element, a potent regulator of ER chaperone levels [38]. Activation of PERK leads to attenuation of global protein translation via phosphorylation and inhibition of the α subunit of eukaryotic initiation factor 2α (eIF2α) [41]. However, phospho-eIF2α does not inhibit the translation of activation transcription factor 4 (ATF4), which is a potent transcription factor for genes involved in amino acid metabolism and transport, oxidation-reduction reactions, and apoptosis [40, 43, 44]. Activated IRE1 has endoribonucleolytic activity that splices X-box binding protein 1 (XBP1) mRNA [45]. The spliced XBP1 transcript is translated and XBP1 translocates to the nucleus where it controls the expression of genes promoting expansion of the ER membrane, protein folding, and degradation of proteins [46]. Unfolded proteins are normally retrotranslocated to the cytoplasm, where they are ubiquitinated and degraded via the proteasome. Cells deficient in IRE1 or XBP1 are defective in ER associated protein degradation [47].

Although all three branches of the UPR are activated by general stress events, the timing, duration and signaling strength of each pathway may vary [48]. Activation of all three branches of the UPR has been described in cancer cells responding to cardiac glycosides *in vitro* [49]. However, inhibition of IRE1 and ATF6 does not affect the expression of ecto-CALR on the plasma membrane [16], suggesting that PERK activity is the key UPR sensor involved in chemotherapy induced ICD. Activated PERK is considered a classical precursor for ICD-associated ecto-CALR expression *in vitro* [16, 19] and ICD *in vivo* [16], but activation of PERK alone does not always result in increased ecto-CALR [50]. This suggests that ER stress is required, but not sufficient, to induce ICD-associated translocation of CALR to the cell surface.

Optimal functioning of ER proteins requires a calcium rich ER environment. There is increasing evidence to suggest calcium leakage from the ER is necessary for ER stress and subsequent surface exposure of CALR [19, 49, 51]. Calcium ionophores mimic the CALR-exposing activity of cardiac glycosides, whose action is blocked by calcium chelators [49]. Reactive oxygen species (ROS) have also been shown to be critical for anthracycline-induced ICD, since treatment with N-acetylcysteine reduced the translocation of CALR to the plasma membrane [16]. However, ROS alone are not sufficient to induce ICD. The direct link between ER stress and ROS in chemotherapy-induced ICD has not been clearly elucidated. Some authors have proposed that ROS generated in the ER lumen is not sufficient to initiate oxidative stress [52, 53]. Intriguingly, calcium leakage from the ER could provide the missing link between ER stress and ROS in chemotherapy-induced ICD. Indeed, calcium release by the ER has been shown to increase mitochondrial calcium loading [54], which activates the Krebs cycle [55, 56], and subsequently promotes generation of ROS from the mitochondrial electron transport chain [57-59]. It is likely that the calcium driven mitochondrial ROS, together with ER lumen generated ROS, may reach the critical threshold required for ICD. In turn, ROS can further increase calcium release by sensitizing ER calcium channels [60]. The positive feedback loop between elevated calcium and increased ROS production may exacerbate ER stress, and could ultimately drive the pre-apoptotic events of ICD. This might explain why disrupting this vicious cycle using either calcium chelators or N-acetylcysteine prevents chemotherapy-induced ICD [16, 49]. To the best of our knowledge, no study has demonstrated the direct link between calcium dysregulation and ROS in chemotherapy-induced ICD, and it is possible that other unknown mechanisms may exist.

**Figure 2:**
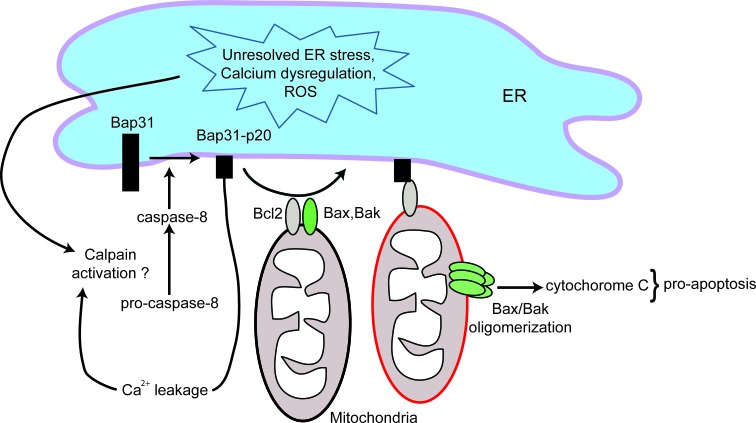
Schematic of the apoptotic module in ICD Unresolved ER stress, calcium dysregulation and elevated levels of reactive oxygen species (ROS) lead to the activation of caspase-8. Activated caspase-8 subsequently cleaves Bap31 to generate a pro-apoptotic p20 fragment. Bap31-p20 interacts with Bcl2, causing its dissociation from Bax and Bak. This allows for the oligomerization of Bax and Bak, leading to disruption in mitochondrial permeability, cytochrome c release and subsequent cell death. The formation of Bap31-p20 fragment also further perturbs calcium homeostasis.

Once ER stress has overwhelmed the adaptive capabilities of the UPR, the “pre-apoptotic module” of the CALR exposure pathway is initiated [16]. ER stress can induce apoptosis via several mechanisms [61], however, only caspase-8-mediated activation has been shown to be essential for ICD [16]. Interestingly, the mechanism of caspase-8 activation in chemotherapy induced ICD remains unknown [16]. Shiga toxin 1-induced ER stress promotes calcium release from ER stores and subsequently leads to the activation of the calcium-dependent protease calpain [62], which leads to caspase-8-mediated cell death. Therefore, it is possible that calpain may be the protease responsible for activating caspase-8 during chemotherapy-induced ICD. Activated caspase-8 subsequently cleaves B cell receptor associated protein 31 (Bap31), an ER-sessile protein [63]. This cleavage generates a pro-apoptotic p20 fragment that interacts with the apoptosis regulator Bcl2 (B cell lymphoma 2) to release sequestered Bax (Bcl2-associated protein x) and Bak (Bcl2 agonist killer 1) [16, 63, 64] (Figure 2). Bax and Bak oligomerize to initiate irreversible events that disrupt mitochondrial permeability, leading to cytochrome c release and subsequent cell death. Bap31 is also a calcium gatekeeper, and cleavage of Bap31 allows leakage of calcium into the cytoplasm, which may further enhance Bax/Bak oligomerization and cytochrome c release [53, 63]. It is important to note that translocation of CALR occurs prior to cleavage of caspase-3 (a terminal event in the intrinsic apoptosis cascade) [16]. Hence CALR translocation is an early event that requires activation of caspase-8 but precedes downstream apoptotic events. Pharmacologic inhibition of caspase-8, or replacement of Bap31 with a non-cleavable mutant, inhibited CALR exposure induced by anthracyclines or oxaliplatin [16]. Similarly, knocking out/down Bax or Bak prevents CALR translocation [16]. Therefore strategies that enhance this apoptotic module may also enhance ICD. Once the ER stress response and the subsequent pre-apoptotic module are complete, the translocation of CALR to the cell surface is initiated. A direct interaction between CALR and ERp57, an ER chaperone that plays important roles in protein folding, MHC loading and quality control of glycoproteins, was shown to be required for the CALR translocation to the cell surface [16]. This process involves actin cytoskeleton-mediated anterograde transport of CALR from the ER to the Golgi apparatus, and subsequent active exocytosis of CALR-containing vesicles [16]. Vesicle associated SNAREs (soluble N-ethylmaleimide-sensitive factor attachment proteinreceptors) (e.g. VAMP-1) and plasma membrane associated SNAREs (e.g. SNAP 23/25) have been shown to be critical for CALR exposure [16].

Extracellular and surface-exposed CALR binds to several cell surface receptors including CD69, CD91, complement component C1q and mannose binding lectin [65]. Ecto-CALR functions as a potent “eat me” signal on apoptotic cells through the binding of CD91 on macrophages and DCs [66]. Similar to CALR, phosphatidylserine (PS) also serves as an “eat me” signal on apoptotic cells, but CALR exposure precedes that of PS [22]. In contrast to CALR, PS mediates clearance of tumor cells without activating an immune response [67]. PS exposure on cancer cells has also been associated with immunosuppression, and antibody-mediated inhibition of PS significantly improves anti-tumor immune responses [68, 69]. CALR binds CD91 on APCs to promote pro-inflammatory cytokines (e.g. IL-6 and tumor necrosis factor) [70, 71]. Hence, recognition of CALR on dying cells is an early event that leads to engulfment of dying tumor cells by APCs and promotes priming of the adaptive immune response. In addition to the “eat me” signals on dying tumor cells, co-stimulatory DAMPs are required to generate an effective anti-tumor immune response (most commonly ATP and HMGB1).

## THE ROLE OF ATP IN ICD

ATP has been widely studied for its role in energy metabolism and autocrine/paracrine cell signaling. Although ATP has a physiologic role in neurotransmission, it can also be released from cells during pathological conditions such as mechanical stress, plasma membrane damage, hypoxia and exposure to cytotoxic agents [72]. Multiple distinct ICD inducers (oxaliplatin, mitoxanthrone, doxorubicin) can cause ATP release from dying tumor cells [21, 72]. Although ATP release could occur through multiple mechanisms, autophagy is thought to be the primary mechanism that sustains high ATP levels in cells undergoing ICD [12].

Currently, it is not clear how autophagy is initiated in chemotherapy-induced ICD. ER stress is initiated early following exposure to inducers of ICD and failure of the UPR results in accumulation of unfolded proteins. Usually the proteasomal pathway is involved in degradation of short-lived proteins [73], whereas the autophagy process is involved in breaking down long-lived or aggregated proteins [74]. In the case where the UPR/proteasomal pathway is overwhelmed by the accumulation of misfolded proteins, excess proteins may form aggregates that trigger autophagy. ER stress can also directly promote autophagy. During ER stress, IRE1 signals through TRAF-2 to activate c-Jun N-terminal Kinase (JNK) and subsequently phosphorylate Bcl2 [33]. This promotes the dissociation of Beclin-1 from Bcl2, an important step in progression of autophagy. In addition, ER stress can also directly regulate the transcription of autophagy related genes [75]. Following ER stress, activation of eIF2α/ATF4 pathway increases the transcription of genes implicated in the formation, elongation and function of the autophagosome (*Atg16l1*, *Map1lc3b*, *Atg12*, *Atg3*, *Becn1*, and *Gabarapl2*) [75-78]. Alternatively, activation of the eIF2α/ATF4 pathway can also upregulate the expression of stress-regulated protein p8 (also known as candidate of metastasis-1) and its downstream target, pseudokinase Tribbles homologue 3 (TRB3) [79, 80]. TRB3 is a known activator of autophagy via inhibition of the Akt/mTOR complex 1 (mTORc1) [33, 80]. Other mechanisms of ER stress-mediated autophagy induction have been reviewed elsewhere [81], however to the best of our knowledge, no study has shown these interactions in the context of chemotherapy-induced ICD. Taken together, ER stresses can upregulate autophagy via multiple independent mechanisms. We hypothesize that in the early stages of ICD, autophagy might be intervening to try and relieve ER stress. In the event that both UPR and autophagy fail to rescue the cell, it is possible that both pathways work together to enhance ICD. If this is true, it suggests a certain threshold or point of no return for the cell exists, perhaps linked to mitochondrial permeability.

Autophagy is a catabolic process that results in bulk degradation of cytoplasmic contents, abnormal protein aggregates, and excess or damaged organelles [82]. Autophagy cargo is sequestered into double-membraned compartments (called autophagosomes) that fuse with lysosomes to degrade their contents and generate raw materials and energy. Although ATP release from cells could occur through multiple mechanisms, autophagy has been shown to be important for optimal release of ATP from dying cells [12]. In a series of elegant experiments, Martins *et al.* [83] demonstrated that pre-apoptotic autophagy is essential in promoting the accumulation of ATP in LAMP1^+^ (Lysosomal-associated membrane protein 1) autolysosomes. However, subsequent translocation of LAMP1^+^ vesicles to the plasma membrane and release of ATP are autophagy independent. The secretion of ATP during ICD was dependent on caspase-mediated opening of pannexin 1 channels. Importantly, autophagy and LAMP1 failed to influence pannexin 1 channel opening, but pannexin 1 was indispensable for the translocation of LAMP1 to the plasma membrane. Hence, it appears that autophagy is the means by which ATP levels concentrate in LAMP1^+^ lysosomal vesicles, and caspase/pannexin-1 dependent lysosomal exocytosis are key in the subsequent release of ATP by cells undergoing ICD [83, 84]. Pharmacologic inhibition or genetic targeting of key components of the autophagosome such as Atg5, Atg7 or Beclin-1 led to a significant reduction in ATP release and limited the immunogenicity of dying cancer cells [12]. Tumor cells deficient in autophagy and ATP production exhibited impaired recruitment of monocytes, macrophages and dendritic cells following therapy [12]. Local injections of ectonucleotidase inhibitors (that block ATP degradation) into tumors was sufficient to rescue extracellular ATP levels, enhance DC and T cell infiltration, and improve chemotherapeutic outcomes [12]. This suggests that the process of autophagy is dispensable, and any source of ATP is sufficient to rescue ICD.

**Figure 3:**
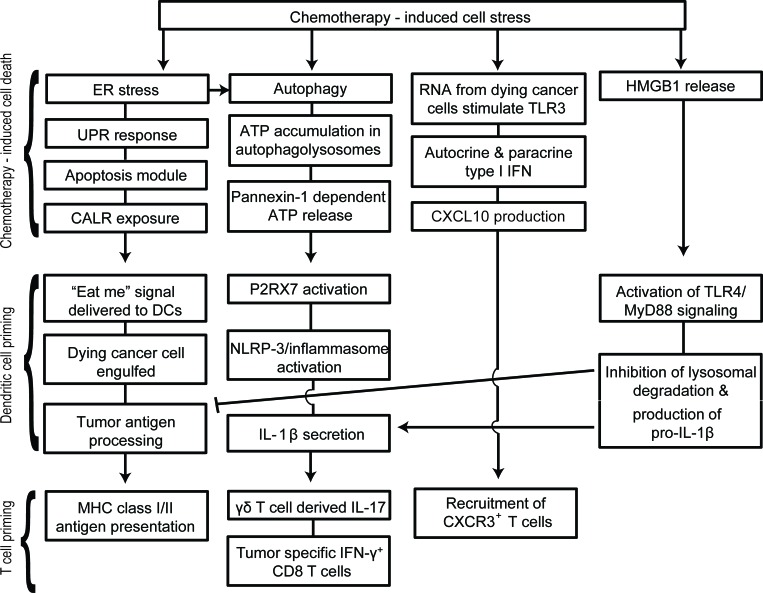
Schematic representation of therapy-induced immunogenic cell death (ICD) Chemotherapy-induced ER stress, autophagy, TLR3 activation and HMGB1 release are key events in immunogenic cell death. Unresolved ER stress leads to the activation of the unfolded protein response, initiation of the pre-apoptotic module and subsequent translocation of CALR. CALR is a potent “eat me” signal for infiltrating DCs. Autophagy plays an important role in accumulation of ATP in phagolysosomes and ATP potently activates the NLRP3/inflammasome pathway in DCs. Following activation of the inflammasome, DCs secrete IL-1β that is essential in the recruitment of γδ T cells and IFN-γ^+^ cytotoxic CD8 T cells. Activation of TLR3 in dying cancer cells also leads to autocrine/paracrine type I IFN production and the subsequent production of CXCL10. This is followed by the release of HMGB1 during the late stages of apoptosis.

Extracellular ATP has the dual effect of attracting immune cells and activating the inflammasome pathway. ATP signaling via P2Y2 receptors on monocytes and DCs induces their recruitment and differentiation in the tumor microenvironment [85-87]. Once naïve immune cells are recruited to tumor sites and have been exposed to “eat me” signals, they require activation signals to increase their anti-tumor activities. ATP signaling via P2RX7 receptors is one of the most potent activators of the NLRP3 inflammasome pathway in DCs and macrophages [85, 88, 89]. Although P2RX7 is expressed on several cell types, ATP primarily acts through P2RX7 on DCs during ICD [21]. The NLRP3 pathway activates the protease caspase-1, which leads to the processing and secretion of mature pro-inflammatory IL-1β and IL-18 [89]. Supporting a key role for the ATP/NLRP3 inflammasome in response to chemotherapy, Ghringhelli et al. [21] demonstrated that P2RX7^−/−^, NLRP3^−/−^, Caspase- 1^−/−^, and IL-1R^−/−^ mice had significantly decreased chemotherapeutic protection against EG7 lymphoma, CT26 colorectal carcinoma and MCA205 fibrosarcoma. Furthermore, using anti-IL-1β blocking antibodies, chemotherapy induced IL-1β was shown to be required for the recruitment of IL-17-producing γδ T cells [87] and generation of IFN-γ-producing tumor-specific CD8 T cells [21]. Interestingly, the recruitment of γδ T cells and production of IL-17 occurred prior to tumor-specific IFN-γ production by CD8 T cells [87]. Therefore mice lacking γδ T cells, IL-17 or IL-17R failed to recruit IFN-γ-producing CD8 T cells. Hence ATP release following cytotoxic therapy can mediate activation of the NLRP3 inflammasome, creating a link between the innate and adaptive immune response. In particular, activation of the inflammasome in DCs leads to the production of IL-1β and subsequent recruitment of γδ T cells and priming of CD8 T cells against tumor antigens (Figure 3).

## THE ROLE OF TLR3 AND TYPE-I IFN IN ICD

**Figure 4:**
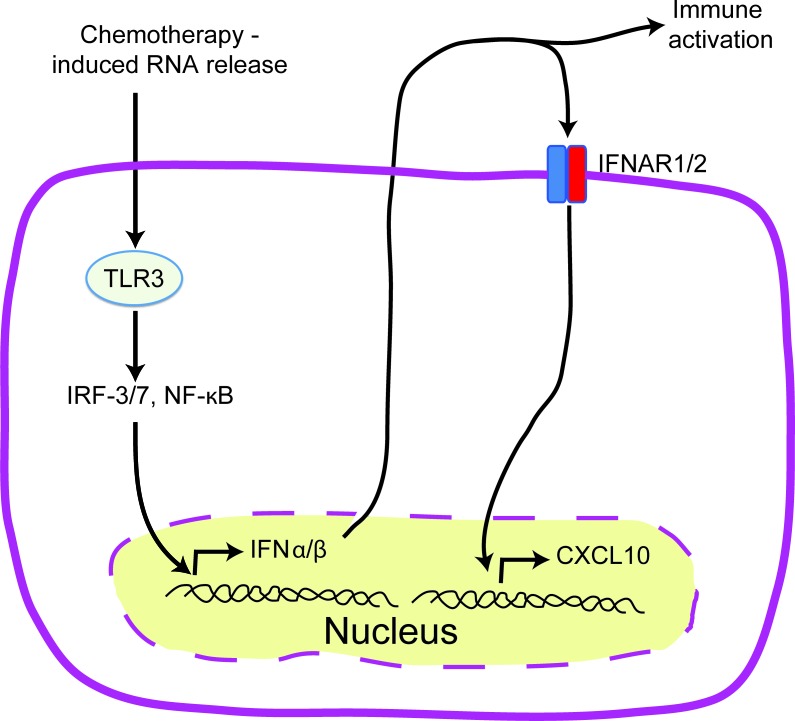
TLR3/IFN-α/β/CXCL10 axis in chemotherapy induced ICD Schematic representation of the events preceding the release of tumor derived CXCL10 in ICD. Treatment with anthracyclines activates TLR3 signaling in cancer cells which leads to the rapid release of IFNα/β by cancer cells. IFNα/β can act in both an autocrine or paracrine manner on neoplastic cells leading to the release of CXCL10.

Ligation of TLRs by microbial ligands is known to trigger well characterized signaling cascades that result in anti-microbial immune responses [90], and occasionally, death of the infected cells [91-93]. TLR3 is a key endosomal pathogen recognition receptor for dsRNA and is required for full induction of type I IFN in anti-viral immune responses [94, 95]. While TLR3 agonists have been shown to cause cell death in pancreatic β cells [96, 97], endothelial cells [98], and cancer cells [99], a novel role of anthracycline induced TLR3 activation has been recently described in ICD [26]. TIR domain-containing adapter inducing IFN-β (TRIF), the only known adaptor protein of TLR3 signaling, can by itself exhibit pro-apoptotic properties [100-102]. Sistigu *et al.* [26] demonstrated that anthracyclines elicit aTLR3 signaling cascade in cancer cells that leads to autocrine/paracrine type I IFN signaling and the subsequent secretion of CXCL10 (Figure 4). Genetically knocking out TLR3 or the IFN-α/β receptor (IFNAR) in cancer cells ablated this protection, an effect that could be reversed by administering recombinant type I IFN or CXCL10 in the respective knockouts [26]. This cascade was essential for the successful vaccination of mice against tumor rechallenge. Consistent with this, a type I IFN signature is a strong prognostic factor for breast cancer patients undergoing anthracycline therapy [26]. The availability of clinical grade TLR3 agonists [103], recombinant type I IFNs [104], and pre-clinical recombinant CXCL10 [105] provide promising avenues for targeted cancer therapies.

## THE ROLE OF HMGB1 IN ICD

HMGB1 is a ubiquitously expressed protein that plays an important role in stabilizing nucleosomes, regulating gene transcription, and DNA repair [106, 107]. In addition to its role in the nucleus, extracellular HMGB1 has key roles in inflammation, cell differentiation, cell migration, and tumor metastasis [108]. HMGB1 is actively secreted by macrophages [109], DCs [110], and NK cells [111] in response to infection or injury. Secreted HMGB1 can promote inflammation by binding the receptor for advanced glycation end products (RAGE), TLR2, TLR4 and TLR9, whereas HMGB1 pro-inflammatory activity can be inhibited by binding CD24 [112-115]. When bound to nucleosomes, HMGB1 can induce a TLR2 mediated humoral responses against the released DNA/histones [116]. HMGB1 can also associate with the chemokine CXCL12 and recruit immune cells to sites of inflammation [117].

Although early reports suggested that HMGB1 was only released from necrotic cells [114], there is increasing evidence showing that HMGB1 can be released during the late stages of apoptosis [115, 118]. In particular, the release of HMGB1 was shown to be required for effective induction of ICD [11]. In this study, the authors vaccinated mice with anthracycline- or oxaliplatin-treated cells one week prior to tumor challenge and demonstrated that antibody mediated blockade of HMGB1 compromised the efficacy of vaccination. Furthermore, protection in MyD88 and TLR4-deficient mice (but not other TLR-deficient mice) was also compromised using this vaccination strategy. These results implicate TLR4 as the receptor for HMGB1 that mediates anti-tumor immune responses via chemotherapy-induced ICD. The activation of TLR4 on DCs in culture was shown to enhance the processing of phagocytic cargo, facilitate antigen presentation, upregulate co-stimulatory molecules and increase intracellular levels of pro-IL-1β (a substrate for the inflammasome pathway) [7, 11, 72]. TLR4 signaling might also prevent the premature lysosomal degradation of engulfed apoptotic debris and potentially preserve tumor-associated antigens for presentation [11, 119]. This is consistent with the observation that peptides that are resistant to lysosomal degradation are more antigenic [119]. Tumor cells deficient in HMGB1 exhibit compromised capacity to induce ICD and anti-tumor immune responses, however, TLR4 agonists rescued the chemotherapy-induced anti-tumor immune responses [27]. Similarly, inhibition of lysosomal degradation using chloroquine enhanced the efficacy of chemotherapy in TLR4^−/−^ mice but not wild type mice [11]. This suggests that TLR4 signaling in DCs and not the specific TLR4 ligand produced by dying tumor cell is important for inducing ICD.

Although chemotherapy-induced HMGB1/TLR4 signaling has primarily been associated with anti-tumor immune responses, HMGB1 has also been shown to promote cancer regrowth and metastasis in cells that survived chemotherapy [120, 121]. Blocking RAGE-HMGB1 interactions significantly reduced tumor burden in both spontaneous and implanted tumor models [121]. Similarly, HMGB1 has been shown to mediate colitis-associated tumors, which can be decreased in incidence and size by anti-HMGB1 antibody treatment [122]. The necrotic cells arising from dextran sulfate sodium-induced colitis may account for the high levels of HMGB1. This pro-tumorigenic role of HMGB1 may be linked to chronic inflammation, which has been associated with tumor development and progression [123]. However, HMGB1 may also have direct deleterious effects on anti-cancer therapy. HMGB1 can directly inhibit the efficacy of DNA vaccines and chemotherapy by interacting with T cell immunoglobulin and mucin domain-containing protein 3 (TIM-3) on DCs and inhibiting the uptake of nucleic acids into endosomes [124].

**Figure 5:**
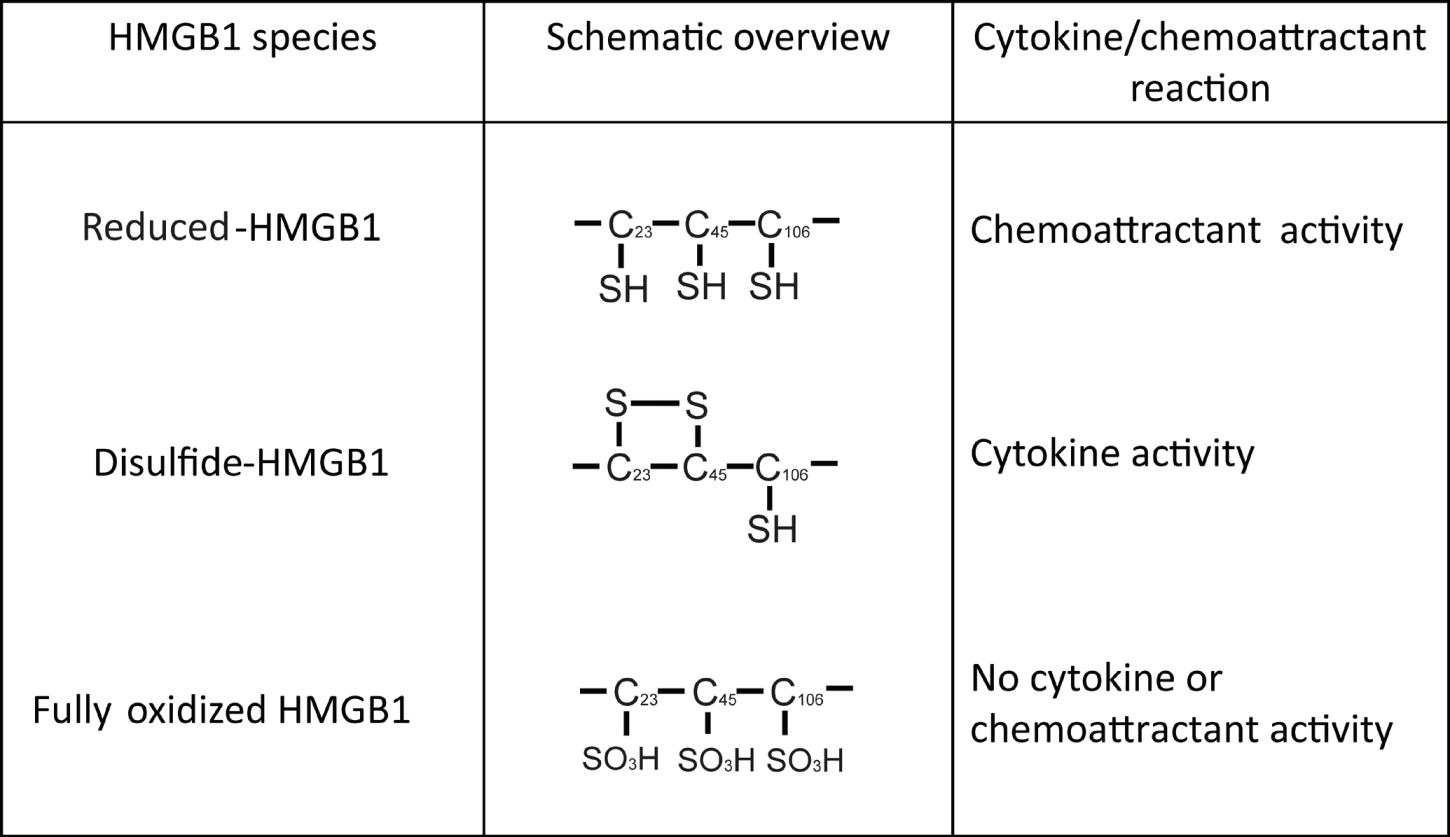
Mutually exclusive forms of HMGB1 The redox dependent cytokine-stimulating and chemoattractant properties of HMGB1.

These conflicting roles of HMGB1 could be attributed to the ability of HMGB1 to switch among mutually exclusive oxidative states (Figure 5). HMGB1 contains three conserved cysteine residues that are sensitive to oxidation; Cys23, Cys45 and Cys106 [125]. When all three cysteine residues on HMGB1 are reduced, HMGB1 binds CXCR4 and acts as a chemoattractant. Formation of a disulfide bond between Cys23 and Cys45 leads to the preferential binding to TLR4 and induces pro-inflammatory cytokine production. Further ROS-mediated oxidation of all three cysteine residues to sulfonates abrogates both activities [126]. Terminally oxidized HMGB1 is associated with increased resistance to chemotherapy [108, 127]. The mechanism by which HMGB1 redox state is regulated in the tumor microenvironment remains unknown, and it is possible that these changes occur either prior to HMGB1 release from the dying cell or following the release of HMGB1 into the tumor microenvironment.

## CONSIDERATIONS FOR ICD IN CANCER IMMUNOTHERAPY DEVELOPMENT

These emerging concepts surrounding chemotherapy-induced ICD bring promises, questions, challenges, and opportunities that need to be further investigated to enhance potential clinical benefit. In particular, the concepts underlying ICD could play an important role in patient-specific chemotherapy selection, tumor-specific therapeutic strategies or dose/sequence selection. In recent years, immune modulation therapy has gained traction as an emerging standard of care for many types of cancers. Strategies for combining immunotherapies with ICD-inducing chemotherapies could potentially lead to enhanced efficacy and diminished toxicities associated with current therapeutic strategies. However, there are currently no well-established protocols for combining immune modulation therapy with ICD-inducing chemotherapies. Several factors that should be considered when combining immunotherapy with ICD-inducing chemotherapy are outlined below.

## CALR “EAT ME” VS CD47 “DON'T EAT ME” SIGNALS

In non-Hodgkin's lymphoma patients receiving autologous DCs loaded with tumor cell antigens (generated by heat shock or irradiation), levels of CALR exposure on cancer cells provided an independent prognostic marker [13]. Similarly, in acute myeloid leukemia (AML) patients receiving anthracycline therapy, the ability of autologous T cells to produce an antigen-specific IFN-γ response was strongly correlated with the tumor associated ER stress (measured by phospho-eIF2α) and ecto-CALR expression [128]. Chemotherapeutics such as cisplatin fail to trigger CALR exposure due to the inability to induce ER stress, but co-administration of ER stressors such as thapsigargin or tunicamycin can correct this defect [28, 30, 129-131]. Patients who receive *bona fide* ICD inducers and fail to upregulate CALR or patients receiving non-ICD-inducing chemotherapies such as cisplatin may potentially benefit from a combination therapy with ER stressors or administration of exogenous recombinant CALR [16, 22, 131, 132]. However, expression of ecto-CALR alone is not sufficient to promote clearance of dying cells, since it is also important that the “don't eat me” signals mediated by CD47 are absent or inhibited [128, 133]. CD47 is a ubiquitously expressed transmembrane protein that binds to SIRPα (signal regulatory protein α) on phagocytic cells, including macrophages and DCs [134, 135], initiating a cell signaling cascade that inhibits phagocytosis [136, 137]. Cancer cells appear to upregulate CD47 as a mechanism of immune evasion [138-140]. Indeed, elevated CD47 expression is strongly correlated with resistance to anti-cancer therapy [141]. Antibody-mediated blockade of CD47 was shown to increase macrophage-mediated phagocytosis of leukemia and solid tumor cells *in vitro* and reduce tumor burden and enhance survival *in vivo* [140, 142]. Therefore, combining CD47 blockade with ICD inducers may theoretically enhance therapeutic outcomes.

## POLYMORPHISMS IN HMGB1, TLR4, AND P2RX7

Retrospective clinical studies show loss of HMGB1 expression in tumors is associated with disease progression [27] and poor survival [143]. In addition, breast cancer patients with a loss-of-function single nucleotide polymorphism (SNP) in the TLR4 gene (Asp299Gly) were more susceptible to relapse from anthracycline based therapy [11]. Similarly, a TLR4 gene loss-of-function SNP (Thr399Ile) in patients with head and neck cancers [144] or colorectal cancers [28] was associated with worse overall survival following anthracycline and oxaliplatin based therapies, respectively. Breast cancer patients responding to neoadjuvant chemotherapy with epirubicin/docetaxel had higher levels of plasma HMGB1 compared to patients who did not respond to therapy [145]. Therefore, deficiencies in HMGB1 or mutations in TLR4 can be an independent predictive factor for therapeutic success, and perhaps these patients could benefit from combination therapies that overcome these defects. As mentioned earlier, the deficiency in antigen cross-presentation and lack of ICD response in TLR4^−/−^ mice could be rescued by systemic administration of chloroquine [11]. Addition of chloroquine to conventional chemotherapy and radiotherapy was shown to improve the mid-term survival of glioblastoma patients [146]. However, the HMGB1/TLR4 status of these patients was not examined in the study.

The clinical relevance of ATP/P2RX7 is best depicted by a loss of function SNP in P2RX7 (Glu496Ala) which lowers the affinity for ATP [147]. This polymorphism decreases ATP mediated IL-1β release by human monocytes, indicative of impaired inflammasome activation [147]. P2RX7 (Glu496Ala) mutations in breast cancer patients receiving anthracycline therapy were associated with significantly lower metastasis free survival compared to patients bearing normal P2RX7 alleles [21]. This is consistent with the pre-clinical observations that disrupting ATP/P2RX7 signaling may affect anthracycline induced ICD and impair response to therapy. Local administration of recombinant IL-1β and IL-12 fully restored anti-tumor T cell responses in P2RX7^−/−^, NLRP3^−/−^ and Caspase-1^−/−^ mice [21], suggesting that the use of biologics may potentially benefit patients that have defects in these pathways. Although ATP release by dying cells is thought to be derived from autophagy mediated accumulation in lysosomes, ATP levels in the tumor microenvironment can also be influenced by the rate of ATP degradation. Extracellular ATP degradation occurs through ectonucleotidases such as CD39 and CD73. CD39 degrades ATP to ADP and AMP, whereas CD73 processes AMP to immunosuppressive adenosine [148]. The role of CD39 and CD73 in tumor progression was illustrated by the development of resistance to immunotherapy and chemotherapy in tumor cells transfected with CD39 or CD73 [12, 149]. In line with this, high expression of CD39 and CD73 was correlated with a poor prognosis in leukemia [150] and colorectal carcinoma [151]. However, CD39 and CD73 expression on immune cells has also been shown promote tumor immune escape [149, 152]. Mice deficient in CD39 and CD73 showed enhanced anti-tumor NK cell [152], and CD8 T cell responses [149], respectively. Collectively, these studies either directly or indirectly implicate a protective role of ATP in tumor control, and suggest that strategies to enhance ATP levels in the tumor microenvironment might enhance therapeutic outcomes. In addition, screening cancer patients for SNPs in TLR4 and P2RX7 could help identify patients who may benefit from additional therapeutic interventions to overcome these mutations.

## MICROBIOME

There is increasing evidence to implicate the microbiome in many physiological processes, and microbial dysbiosis is associated with pathological outcomes [153]. In particular, the gut microbiome can directly influence cancer therapy by regulating host innate and adaptive immune responses [154, 155]. Germ-free mice or mice pre-treated with antibiotics exhibit significantly reduced responses to anti-cancer chemotherapy [154, 155]. Oxaliplatin, a known inducer of ICD, was shown to eradicate most subcutaneous EL-4 tumors and prolong survival in normal mice, but not in antibiotic treated or germ-free mice [154]. Similarly, cyclophosphamide was shown to protect mice from tumors by disrupting the integrity of the gut mucosa, resulting in colonization of secondary lymphoid organs by Gram-positive bacteria and a subsequent Th17-dependent anti-tumor response [155]. Antibiotic therapy was shown to significantly hamper the protective effects of cyclophosphamide. In addition to influencing the adaptive immune response, commensal microorganisms may play a role in the enhancement/inhibition of chemotherapy induced TLR3/IFN/CXCL10 responses. Given that TLR3 has been well studied in innate anti-microbial responses against intracellular pathogens, it is possible that microbes within the tumor microenvironment may directly influence TLR3 responses. Further studies will be required to completely delineate the role of the microbiome in chemotherapy induced ICD in both pre-clinical and clinical settings.

## PATIENT IMMUNE STATUS

One of the hallmarks of cancer progression is the induction of immunosuppression, which allows the tumor to evade detection and/or elimination by the immune system [123, 156]. Tumors suppress the adaptive immune response at the level of antigen presentation by downregulating expression of tumor antigens, antigen processing machinery, and MHC class I and II molecules [157, 158]. In addition, tumor cells can drive the expansion of immunosuppressive myeloid derived suppressor cells (MDSCs), a heterogeneous population of undifferentiated myeloid cells [159]. MDSCs can directly suppress T cell responses, and indirectly promote immune suppression through the induction of FoxP3^+^ regulatory T cells (Tregs) [160-164]. Given that the benefits of ICD require antigen presentation by DCs and a functional T cell response [6, 12, 21, 22, 26, 165], patients that have suppressed immune systems may exhibit reduced responses to chemotherapeutics that induce ICD. These patients may benefit from combination therapies that combine inducers of ICD with immunostimulation or targeting of immunosuppressive populations. One potential strategy would be to combine ICD inducers with immunostimulatory antibodies that directly modulate immune functions by enhancing stimulatory signals or blocking inhibitory signals. These include anti-CTLA-4 (antagonizes cytotoxic T lymphocyte antigen 4) [166], anti-PD1 (blocks programmed death protein 1) [167], anti-TIM3 (blocks T cell immunoglobulin and mucin domain-containing protein 3) [168], CD40 agonists [169] and OX40 agonists [170, 171]. Although a detailed description of these agents is beyond the scope of this review, several pre-clinical studies have demonstrated enhanced protection when these compounds were combined with cyclophosphamide [172, 173], a known inducer of ICD. An alternative approach would be to use immunostimulatory cytokines to complement ICD inducing chemotherapies. Oxaliplatin used in combination with IL-12 was able to eradicate pre-existing metastatic colorectal cancer and protect from tumor recurrence in a murine model [174]. This combination strategy was shown to significantly increase the ratio of CD8 T cells/Tregs and the ratio of CD8 T cells/MDSCs within the tumors. In addition to ICD-inducing activity, doxorubicin has been shown to directly deplete and/or impair the immunosuppressive function of MDSCs [175]. Paradoxically, some chemotherapies that induce ICD can also induce suppressor cells that can inhibit immune responses [176-178]. Therefore, the immunological profile of the patient receiving ICD-inducing chemotherapy needs to be taken into consideration when designing a patient's chemo-immunotherapy treatment.

The immune system is not exempt from the potential cytotoxicity or cytostatic effects of chemotherapy. Understanding how different chemotherapies directly influence the immune system, may enable us to strategically target tumor cells and manipulate the immune response. Cytotoxic chemotherapy (at high doses) is generally non-specific and targets all proliferating cells, including lymphocytes. While long-term lymphodepletion may be detrimental [179], short-term lymphodepletion may have beneficial effects in cancer therapy [180-182]. First, lymphodepletion results in the elimination of immunosuppressive cells such as Tregs, which have been shown to inhibit anti-tumor immune responses and promote peripheral tolerance [183]. Second, lymphodepletion triggers a resetting of the immune system which is characterized by IL-7 and IL-15 driven homeostatic proliferation of lymphocytes [180]. This homeostatic proliferation provides a critical window of opportunity to skew the immune response towards a specific antigen through vaccination or adoptive transfer of antigen-specific lymphocytes [181, 184]. In a pre-clinical lung cancer model, chemotherapy-induced lymphopenia prior to vaccine administration was shown to promote expansion of effector T cells relative to Tregs [184]. Therefore, these specific situations would provide rational justification for using high doses of chemotherapy to strategically deplete bulk lymphocytes and create a space for reshaping the tumor specific immune response. However, when considering the potential benefits of chemotherapy induced ICD, it is essential that the patient's immune system is able to respond to the dying cell. Recently, the focus has now switched to metronomic low dose chemotherapy, which has been shown to selectively target Tregs and MDSCs without causing gross lymphodepletion [185, 186].

## ANTIGENICITY AND THERAPEUTIC REGIMEN

There are hundreds of potential tumor antigens in any particular tumor [187]. These tumor antigens can broadly be classified as tumor-associated antigens (TAAs) (also expressed on normal cells) or tumor-specific antigens (TSAs) (neo-antigens). TAAs are more abundant than TSAs, but TSAs are generally better at inducing immune responses since they are recognized as non-self. When chemotherapy induces tumor cell death, these antigens are released by dying cells and are taken up by APCs and presented to T cells [188, 189]. Treatment with 5-fluorouracil selectively upregulates the expression of TAAs such as cancer-testis antigen and carcinoembryonic antigen, hence increasing the antigenicity associated with therapy [190]. In transplantable mesothelioma tumor models expressing ovalbumin as a model antigen, the tumor classically elicits an immune response against the dominant epitope SIINFEKL [191]. Treatment with chemotherapeutics was shown to broaden the range of tumor antigens recognized by cytotoxic CD8 T cells [191]. However, co-administration of IL-2 at the same time as chemotherapy completely abolished this phenomenon of “epitope spreading” and refocused the immune response towards the dominant SIIFENKL epitope [191]. This finding suggests that administering immunotherapy too close to ICD-inducing chemotherapy may have negative effects on range of antigens recognized. Consistent with a role in antigen diversification, dacarbazine administration 1 day prior to peptide vaccination in melanoma patients significantly increased the antigenic repertoire of T cells and induced greater tumor reactivity, compared to vaccine therapy alone [192, 193]. In a pilot clinical study examining the safety and feasibility administering cyclophosphamide 7 days prior combined treatment with GM-CSF, pegylated IFN, and DCs loaded with autologous tumor lysate, cyclophosphamide pre-treatment increased levels of IL-12p70, NK cell cytotoxicity, and T cell reactivity to tumor antigens [194]. This study also found cyclophosphamide pre-treatment reduced the frequency of Tregs to levels observed in healthy individuals. Furthermore, pre-clinical studies examining the effect of administering cyclophosphamide before or after vaccine therapy, found that cyclophosphamide administered 1 day before vaccine therapy enhanced anti-tumor immunity [195, 196]. In contrast, administration of cyclophosphamide after vaccine therapy had inhibitory effects on the antigen-specific responses. Therefore, appropriate sequence and timing of treatment play an important role in influencing antigenicity and subsequently determining the outcome of chemo-immunotherapy.

## CONCLUDING REMARKS

Chemotherapy has long been perceived as the practice of using chemicals to either limit the proliferation or cause immunogenically silent death of cancer cells. Based on this presumption, most pre-clinical work was done in immunodeficient mice and without consideration for the role of the immune system in drug efficacy. Recent advances have challenged this old way of thinking, as the immune system has been shown to play key roles in tumor control mediated by chemotherapeutics. In particular, this concept of “dying the right way” has shifted the focus back to the immune system. Therefore, immunologic parameters are slowly gaining recognition as important therapeutic biomarkers for patients enrolled in clinical trials [7, 188, 197]. It is important to note that while ICD is an important component of chemotherapy-associated anti-cancer immune responses, ICD is not the only way chemotherapeutics facilitate an immune response against cancer [4, 7]. Understanding the concepts and mechanisms that underlie chemotherapy-induced ICD may help shed light on new strategies for combining chemotherapy with novel immunotherapy approaches.
